# Coalescent angiogenesis—evidence for a novel concept of vascular network maturation

**DOI:** 10.1007/s10456-021-09824-3

**Published:** 2021-12-14

**Authors:** Bianca Nitzsche, Wen Wei Rong, Andrean Goede, Björn Hoffmann, Fabio Scarpa, Wolfgang M. Kuebler, Timothy W. Secomb, Axel R. Pries

**Affiliations:** 1 Charité – Universitätsmedizin Berlin, corporate member of Freie Universität Berlin, Humboldt-Universität zu Berlin, and Berlin Institute of Health, Institute of Physiology, Berlin, Germany; 2 German Center for Cardiovascular Research (DZHK), Partner site Berlin, 10117 Berlin, Germany; 3 Department of Information Engineering, University of Padua, Padua, Italy; 4 Department of Physiology, University of Arizona, Tucson, AZ 85724 USA

**Keywords:** Coalescent angiogenesis, Capillary mesh, Tissue islands, Intussusception, Splitting angiogenesis, Sprouting angiogenesis, Chorioallantoic membrane (CAM), Intravital microscopy

## Abstract

Angiogenesis describes the formation of new blood vessels from pre-existing vascular structures. While the most studied mode of angiogenesis is vascular sprouting, specific conditions or organs favor intussusception, i.e., the division or splitting of an existing vessel, as preferential mode of new vessel formation. In the present study, sustained (33-h) intravital microscopy of the vasculature in the chick chorioallantoic membrane (CAM) led to the hypothesis of a novel non-sprouting mode for vessel generation, which we termed “coalescent angiogenesis.” In this process, preferential flow pathways evolve from isotropic capillary meshes enclosing tissue islands. These preferential flow pathways progressively enlarge by coalescence of capillaries and elimination of internal tissue pillars, in a process that is the reverse of intussusception. Concomitantly, less perfused segments regress. In this way, an initially mesh-like capillary network is remodeled into a tree structure, while conserving vascular wall components and maintaining blood flow. Coalescent angiogenesis, thus, describes the remodeling of an initial, hemodynamically inefficient mesh structure, into a hierarchical tree structure that provides efficient convective transport, allowing for the rapid expansion of the vasculature with maintained blood supply and function during development.

**Supplementary Information:**

The online version contains supplementary material available at 10.1007/s10456-021-09824-3.

## Introduction

The generation of blood vessels is a vital process for embryonic development and organ growth, as well as in various pathophysiological conditions such as wound healing, ischemic heart disease and cancer. Two main processes for vascular development are recognized: vasculogenesis and angiogenesis. Vasculogenesis occurs during embryonic development, when the so-called primary vascular plexus is generated by differentiating mesodermally derived angioblasts. This de novo synthesis results in lumenized vascular structures or capillary meshes that generally lack hierarchical organization. In contrast, angiogenesis refers to the generation of new blood vessels from pre-existing ones [1] and supports the expansion of the primary vascular plexus into a more mature vascular network [2, 3]. Two distinct modes of angiogenesis have been described: In sprouting angiogenesis (SA), vessel outgrowth from existing vessels is initiated by specialized endothelial cells termed tip cells. Migration and proliferation lead to an elongation of the initial tip which may eventually connect, or anastomose, with another vessel to form a perfused vascular branch [4, 5]. SA is prominent in situations where perfusion is low and hypoxia is present such as during de novo generation of tissues in embryonic development or wound healing, and is primarily regulated by angiogenetic signals related to local hypoxia [6, 7].

In contrast to SA, intussusceptive angiogenesis (IA) or splitting angiogenesis is a non-sprouting mechanism, first described by Burri et al. for the development of the pulmonary vasculature in postnatal rats [8]. In IA, transluminal endothelial pillars form within a blood vessel and progressively split it into two daughter vessels. These transluminal pillars are formed by evaginations of endothelial cells, creating contact zones between opposite capillary walls. This process allows rapid expansion of a capillary network while maintaining perfusion and, thus, tissue function [9–11] and has since been observed in many tissues including skeletal muscle and kidney [12, 13] as well as in pathological conditions such as COVID-19 [14]. Generation of new blood vessels by this mechanism has also been termed intussusceptive microvascular growth. In contrast, intussusceptive arborization and intussusceptive branching remodeling [15] involve the reorganization of an existing capillary bed via expansion or pruning of vessel branches to optimize branching geometry and hemodynamics in existent vascular trees [16–18].

In addition, a process of vascular fusion was described in the context of blood vessel generation [19–21]. Under physiological conditions all three processes can occur in parallel to meet the functional needs of a vascular bed [22–25].

Sprouting angiogenesis has been intensively studied using intravital imaging approaches, such as the zebrafish model [26, 27]. This has led to substantial knowledge of the underlying basic physiological and molecular mechanisms as well as the cellular dynamics and time courses especially for embryonic development [28, 29]. Due to a paucity of appropriate model systems, such information is less readily available for intussusceptive angiogenesis and the generation of vascular beds in solid organs, including the lung. Consequently, our insight into the basic mechanisms by which such existing capillary beds expand and grow is still scarce. In-depth understanding of these processes requires studies delineating the morphological dynamics of IA vessel generation – similar to those previously reported for SA—to analyze the angiogenic processes and their interaction during organ growth (e.g., of pancreas, lung, or kidney) and in embryonic development.

The chick chorioallantoic membrane (CAM) presents a versatile in vivo model for the study of angiogenesis and vascular adaptation [30] as it facilitates optical access to a developing vascular system in a transparent, two-dimensional layout. Importantly, the CAM allows for extended observation periods for up to 1–2 days, permitting to follow the successive steps of angiogenic development over time [31]. In the fertilized chicken egg, the CAM is created through fusion of the allantois and the chorion from day 3 of embryonic development and its development continues until embryonic day 13 [32]. The CAM serves as the respiratory organ of the embryo [33], and as such is characterized by functional similarities to the human lung, including the transport of deoxygenated blood in arteries and oxygenated blood in venular vessels as well as similar regulatory mechanisms such as vasoconstriction in response to hypoxia [34]. The CAM thus potentially provides a unique model to study the IA in a longitudinal manner and was thus utilized here to delineate the spatio-temporal dynamics of IA as the basis for future mechanistic analyses. In the present study, intravital microscopy of the CAM over a period of 33 h was used to monitor rearrangement and growth of blood vessels and the capillary mesh. Unexpectedly, these observations revealed evidence that mature vessel trees are generated by a previously unrecognized mode of angiogenesis, which is distinct from intussusception and which we termed here “coalescent angiogenesis”.

## Materials and methods

### Ex ovo CAM model

Fertilized specific pathogen-free eggs of white leghorn chicken (Gallus gallus, VALO BioMedia GmbH, Cuxhaven, Germany) were incubated at 37.5 °C and 82% humidity. On day 3 of embryonic development (ED), the eggs were opened under aseptic conditions and the content was transferred into a plastic tissue culture dish (TPP, Trasadingen, Switzerland) as described earlier [35]. The chicken embryos were then again incubated and continued developing in petri dishes until embryos reached Hamburger-Hamilton stage (HH) 36 on ED 11. For intravital microscopy, chicken embryos were placed on a custom‐built temperature controlled (38 °C) and humidified stage [35].

### Scanning protocol

At Hamburger-Hamilton stage 36, the CAM is fully developed and has reached its final location at the surface of the allantoic sac beneath the eggshell. Areas-of-interest in the central parts of the CAM microvascular network were scanned by intravital video microscopy for up to 33 h in 5–7 h intervals. Imaging and video recording was performed on an intravital microscope (Transillumination, Axiotech Vario, Carl Zeiss, Jena, Germany), equipped with both a CMOS camera (Sony ICE 6000, Tokyo, Japan, 1920 × 1080 pixels) and a high-speed camera (MotionBLITZ EoSens mini1-1, MC 1370, 1280 × 1044 pixels, Mikrotron GmbH, Unterschleissheim, Germany). First, an overview video of about 10 s was recorded (2.5x, N.A. 0.085, Zeiss, Germany, field of view (FOV) approximately 7385 × 4154 μm) with a frame rate of 50 fps for a randomly selected area-of-interest. Then 2—4 higher magnified FOV (approximately 2300 × 1200 μm) within the higher magnified FOV were recorded (10x, N.A. 0.22, Leitz, Germany) for at least 4 s. FOVs were chosen that contained the distal end of a draining vessel connected to the capillary mesh. Between the individual scanning procedures, chick embryos were returned to the incubator and maintained under the conditions described.

### Vessel imaging

Image processing of intravital video recordings (4 s) was applied by analysis of intensity information for each pixel from consecutive image frames to enhance the contrast between vascular and extravascular areas as described previously [35–37]. In brief, the image stack of the video recording was first corrected for movement artifacts in the image plane. To this end, horizontal displacement was analyzed by two-dimensional image correlation of consecutive image frames using a defined region of interest (reference square, 256 × 256 or 512 × 512 pixels, respectively) with high image contrast on the first frame of the video. All frames of the video sequence were then aligned according to the displacement of the reference square relative to the first frame.

After movement correction, the standard deviation (SD) of intensity for every individual pixel was calculated over all image frames of the video sequence and normalized for the entire image to the available dynamic range. The standard deviation of intensity relates only to the extent of changes but not to their frequency. The source for changes in image intensity at a given pixel after effective movement correction is light absorption by hemoglobin during the passage of red blood cells. Due to the particulate nature and size of red blood cells, this is strongest in the smallest microvessels or close to the wall in larger conduits. Consequently, SD images allow sensitive differentiation of the lumen of perfused vessels, even very small ones, with high SD values from neighboring extravascular tissue with low SD values. In turn, SD images do not faithfully represent blood flow, requiring a different approach to visualize perfusion.

### Perfusion imaging

**Fig. 1:**
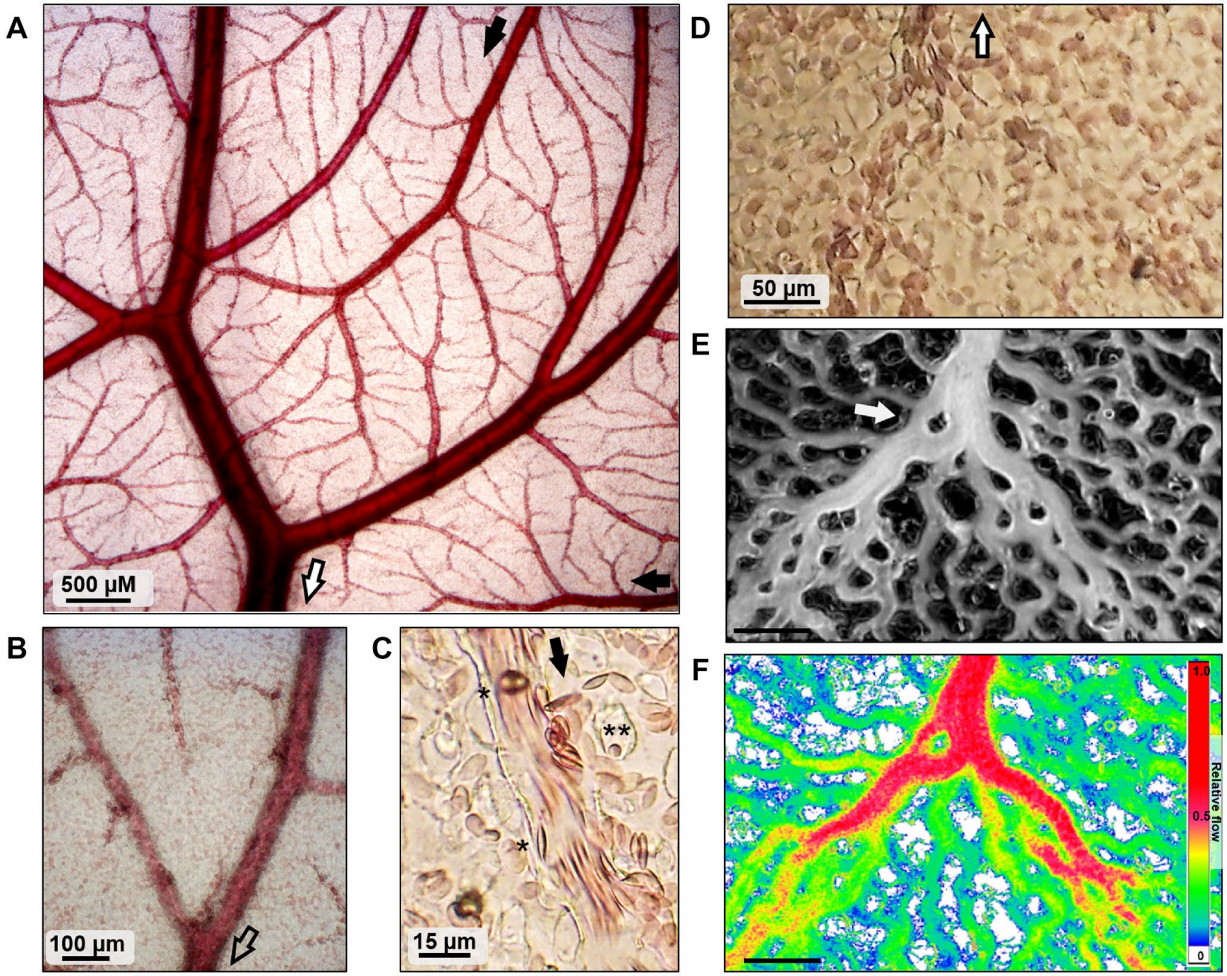
Imaging of the microvascular network of the chick chorioallantoic membrane (CAM). **A-C** Representative intravital micrographs of CAM vascular networks. **A** Overview. Blood flow direction is indicated for major arterial (filled arrows) and venous (open arrows) vessels. **B** Higher magnification of a venous vessel junction. **C** Close-up of a small arteriole (arrow) and capillary mesh with moving red blood cells (*) and tissue islands (**). Structures are difficult to identify from individual still micrographs, but become apparent in dynamic video recordings (see Supplemental Material 1). **D-E** Images of a CAM section before and after image processing. **D** Original image from a 20-s intravital video recording. Only the largest outflow vessel (arrow) is faintly visible. **E** Vessel imaging: standard deviation image (SD) showing vessel structures. High brightnessss corresponds to wide variation of light intensity over time caused by red cell movement. Black regions correspond to tissue islands. In these vessel images, the entire capillary network is visible, including tissue islands and emerging vessel structures (white arrow). **F** Perfusion imaging: pseudo-color image of the sum of intensity changes over time. The image shows preferential flow pathways from the lower left and lower right. In contrast, the upper right quadrant exhibits more evenly distributed flow. The vascular flow pattern corresponds to the vascular morphology in panel E

To allow for semi-quantitative assessment of local microvascular perfusion, an alternative algorithm was used which is sensitive to the total amount of intensity changes for each pixel. To this end, the cumulative amount of intensity changes over the entire recording period was calculated for each pixel and normalized for the entire image to the available dynamic range. The resulting values were converted into pseudo-colors. This algorithm determines the product of amplitude and frequency of intensity changes, which is related to the number of passing red blood cells per time. As such, the pseudo-colors in the resulting images yield a semi-quantitative representation of local perfusion in the microvascular network. However, this algorithm is less well suited for the discrimination of perfused vessels from the tissue as seen by a comparison of panels E and F in Fig. 1. This is due to small but frequent changes in local brightness also in non-perfused areas due to minute tissue movements which cannot be corrected by alignment procedures or by changes in the illumination pattern or focus of the microscope. As a consequence, the integrated intensity change algorithm results in small but positive values also for some avascular tissue areas. The reported perfusion equivalents are meaningful only in areas where the SD algorithm evidences the presence of perfused vessels. Due to normalization, both vessel and perfusion images show only relative levels for a given video sequence, and not quantitative or absolute values.

### Segmentation

A segmentation approach was developed to allow for automated extraction of morphological data from SD images. The original image was convolved with a Gaussian kernel to enhance the intensity difference between pixels corresponding to vessels (white and elongated structures) and tissue islands (dark and circular objects), respectively. The enhanced image underwent a double threshold operation: a global threshold was applied to extract the main vessels and a local threshold was applied to extract the small and secondary vessels. The final vessel segmentation was obtained by the sum of the two threshold operations. Skeletonization was applied to identify the vessels centerline, and morphological operations (erosion and dilation) were used to extract the tissue islands. The skeletonization approach used in the present study extracts the centerline while preserving the topology and Euler number of white objects in a black background. The standard function of Matlab software (MathWorks, Natick, MA) was used for the skeletonization [38]. For this approach, a total of 355 regions of interests (ROI) were selected in n = 4 chicken eggs with a nearly equal number of arterial (117), venous (118) and capillary mesh regions (120).

### Area dynamics

To assess area development of defined CAM sections over time, CAM regions were selected in overview images (2.5 × magnification) that featured recognizable vascular landmarks. For each region, four landmarks (e.g., vessel junctions or branch points) were selected in the image taken at the first time point. These markers, representing the corners of the test region, were identified in all subsequent images, thus allowing to track changes in CAM area.

### Statistical analysis

If not stated otherwise, each CAM experiment was independently reproduced four times (n = 4 CAMs). Correlation coefficients R^2^ were calculated for 120 ROIs (n) from 4 CAMs (N).

## Results

Figure. 1A shows CAM vascular patterns as seen in bright-field intravital microscopy. Arterial and venous trees are arranged in an interdigitating pattern and exhibit a clear hierarchical organization and diameter distribution. Capillary structures are difficult to assess on still bright-field images (Fig. 1B, C) but become apparent in video sequences due to the movement of red blood cells (Supplementary Material 1). Image processing of such sequences allows for differentiation between vascular and non-vascular areas (Fig. 1D, E). The resulting images show that capillaries are organized in polygonal isotropic meshes, with capillaries encircling non-perfused tissue islands and link to feeding or draining vessels via areas with enlarged capillary patterns. These observations are supported by additional image processing which revels perfusion images as described and shows semi-quantitative flow patterns in the vascular network (Fig. 1F). The algorithm permits adequate visualization of the local perfusion in bigger blood vessels but not of the flow of the boundary regions of the microvascular blood vessels including the tissue islands.

**Fig. 2:**
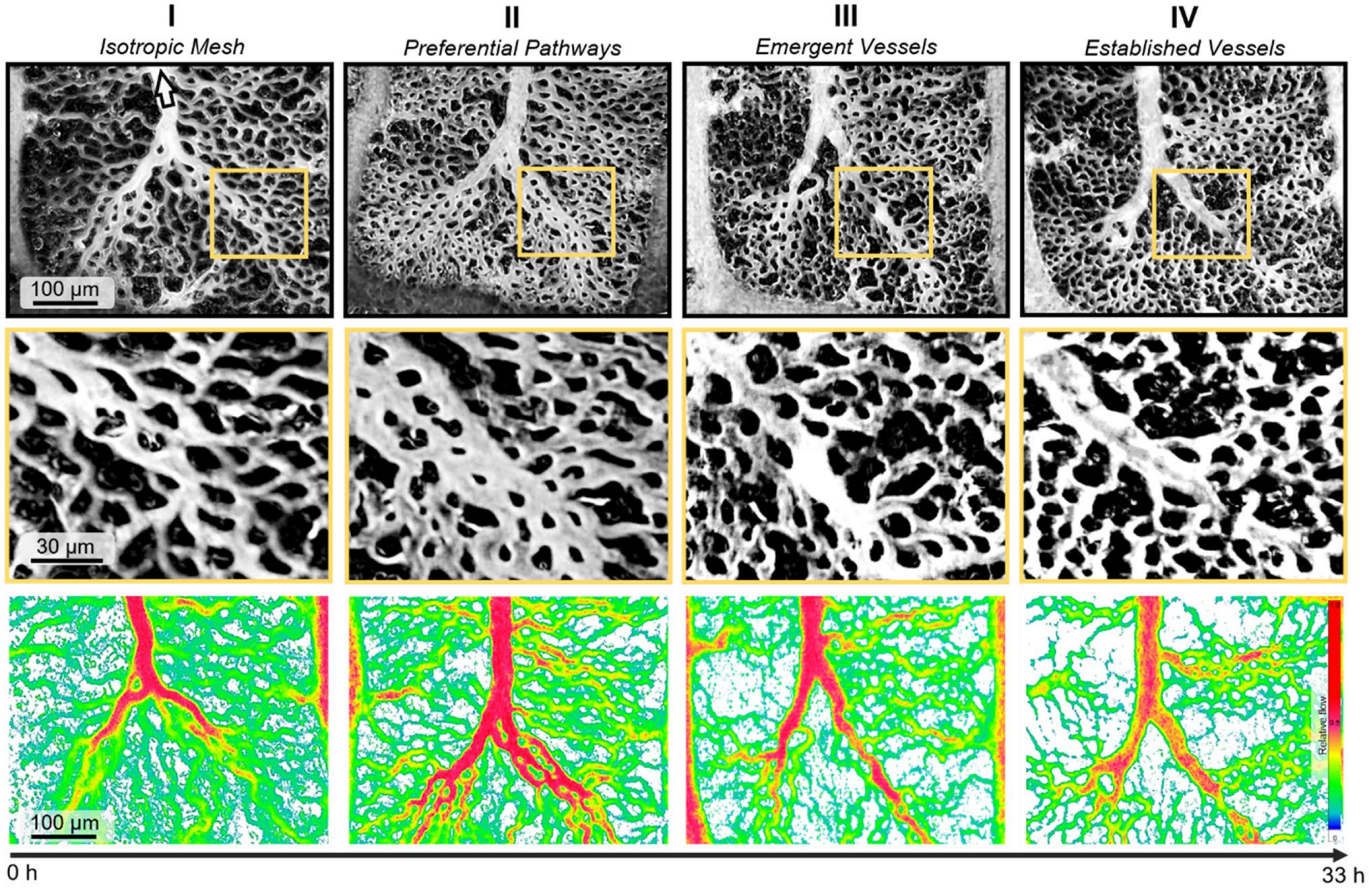
Four main phases of coalescent angiogenesis (CA) from a predominantly isotropic capillary mesh (Phase I) to a hierarchical vascular network (Phase IV) can be distinguished. Flow direction in the main vein is indicated by arrow. Upper panels: Vessel imaging sequence for a CAM microvascular network over a period of 30 h. Middle panels: Close-up of capillaries and tissue islands in the distal vascular network depicting the four different phases of coalescent angiogenesis. Lower panels: Corresponding perfusion images at low magnification showing the relative flow in blood vessels with minimum and maximum intensities. With increasing maturation, relative flow values in the capillary regions decrease in proportion to the high flow in the bigger vessels. Colors in each individual panel are normalized to span the full range. The color scale indicates relative flow

To follow angiogenetic processes in the CAM, time series of selected CAM areas were repeatedly scanned over up to 33 h (Fig. 2). The progression from capillaries into a definitive venule can be differentiated into four phases of blood vessel development. The process starts from a predominantly isotropic capillary mesh in which capillary diameters, length and perfusion exhibit relatively narrow distributions (phase I, isotropic mesh). Within this mesh preferential pathways develop, which are characterized by increased vessel diameter and perfusion (phase II, preferential pathway) with a tendency for a reduction in capillary density in adjacent areas. In phase III (emergent vessel), parallel capillaries coalesce to form a single vessel structure constituting a structural pathway. In this process, tissue islands initially located within the preferential pathway progressively disappear (Fig. 2, middle panels). The last phase (phase IV, established vessel) is characterized by the presence of a definitive vessel with few side branches that now forms an integral part of a hierarchical vessel tree. Microscopic investigation at different focus levels shows that a capillary mesh is partially re-established above the new vessel, which sinks to a slightly lower level toward the yolk sac (see Supplementary Material 2). Essential morphological and hemodynamic properties of the observed phases are summarized in Table 1.

**Table 1:** Characteristics of the different phases of coalescent angiogenesis

	Phase I	Phase II	Phase III	Phase IV
	Isotropic mesh	Preferential pathway	Emergent vessel	Established vessel
Mesh	Uniform, regular	Preferred flow pathways	Separated from vessel	Re-established above new vessel
Vessel wall	Not visible	Not visible	Partly visible	Visible
Perfusion pattern	Homogeneous	Higher flow in pathway	Higher flow in vessel	Highest flow in new vessel
Tissue islands	Polygonal, relatively even size distribution	Rearranged along preferred pathway, small within pathway	Reduced to tissue pillars within vessel	Eliminated within vessel, enlarged in adjacent regions
Blood vessel diameter	Homogeneous capillary diameters	Diameter increase in preferential pathway	Increased diameter in emergent vessel	Further increase in diameter in established vessel

**Fig. 3:**
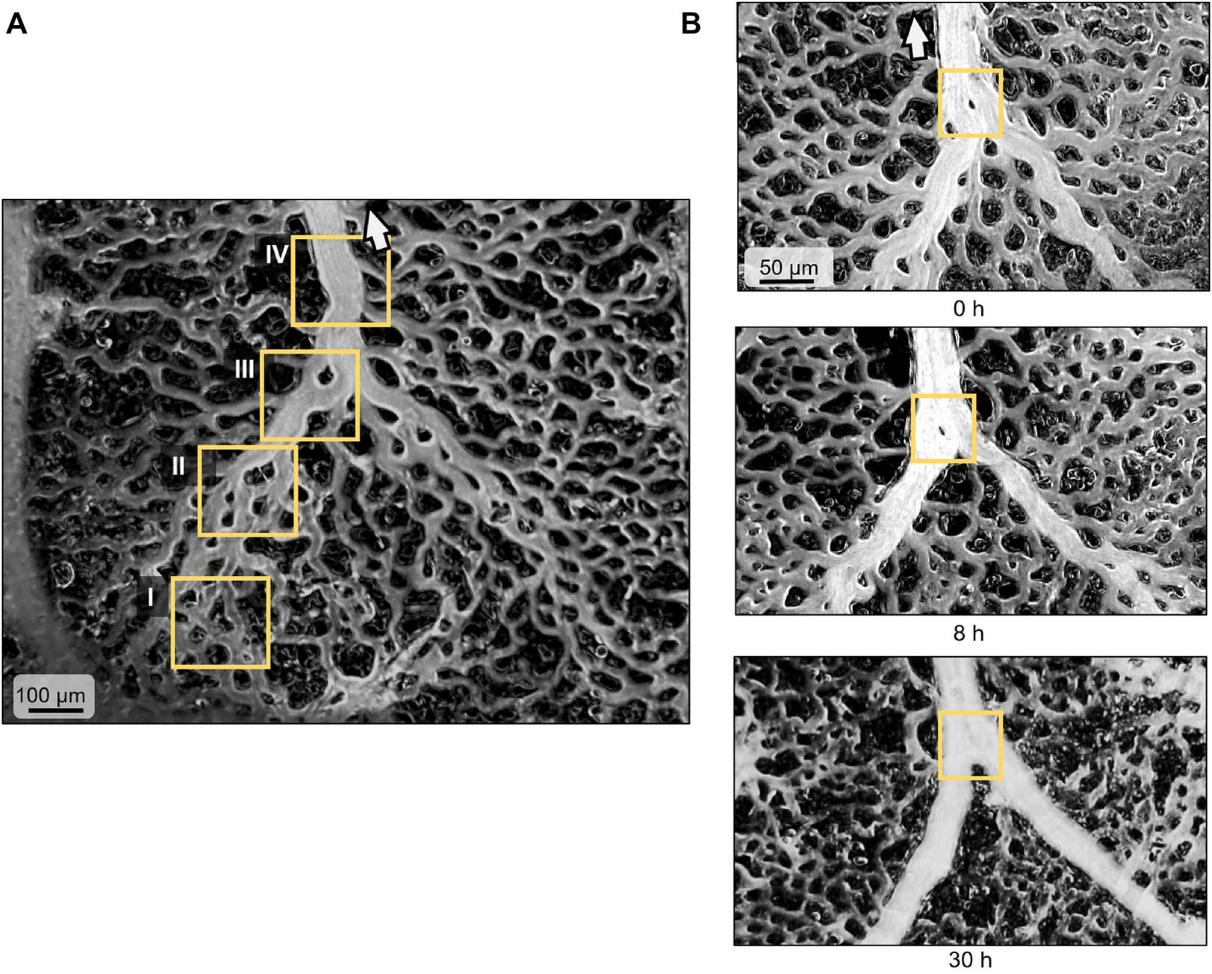
**A** Simultaneous presence of different coalescent angiogenesis phases (yellow squares) along the length of a CAM vessel. I: isotropic mesh; II: preferential pathway; III: emergent vessel; and IV: established vessel. **B** Tissue island at a venular convergence (yellow squares) at three successive time points. The tissue island disappears over the course of the remodeling process, showing reverse intussusception. See Supplemental Material 2 for additional images of this process

These sequential phases of coalescent angiogenesis can not only be observed over time at a given location but are also evident at one single time point along the length of a branching vessel tree, with the early phases corresponding to distal portions of the tree and later phases to its proximal portion (Fig. 3A). Following the vessel tree from distal to proximal, phases I to IV can be clearly distinguished. In the frame corresponding to phase III, a single remnant tissue island can be seen within the coalescent vessel. Such structures were regularly observed. From an individual picture, this structure could be interpreted as a developing tissue pillar, according to the concept of intussusceptive angiogenesis. As shown in the time series (Fig. 3B and Supplemental Material 2), however, these structures actually represent tissue islands that are eliminated in the process of coalescent angiogenesis, rather than newly generated intravascular pillars as would be postulated in intussusceptive angiogenesis. As such, coalescent angiogenesis presents a novel mechanism of network organization that may be adequately described as inverse intussusception.

As shown in the supplemental material (S-Figure S1 and S-Figure S2), there was no significant change of net area and capillary density in isotropic meshes in the central parts of the CAM microvascular network investigated in the present study. Thus, the observed processes of coalescent angiogenesis occur on the background of a constant tissue area and stable capillary topology.

## Discussion

Vascular networks in growing tissues must develop rapidly to meet increasing oxygen and nutrient demands. After the initial formation of vessels by vasculogenesis, the enlargement of a vascular system requires angiogenesis [5] with concomitant vascular remodeling and pruning to achieve hemodynamically efficient network structures [25, 39]. However, information regarding the dynamic steps of intussusceptive angiogenesis is lacking, especially in tissues undergoing fast growth or development. For such conditions, as in the growing lung, skeletal muscle or liver, intussusceptive angiogenesis (IA), i.e., division of pre-existing vessels by newly emerging tissue pillars, has been proposed as a central mechanism [13]. Previous studies have reported intussusception for both single capillaries of the CAM and in the development of larger blood vessels [10, 16, 40, 41]. Such studies were, however, usually performed by analysis of structurally fixed blood vessels or using microscopic observations at a single time point and hence, lack any temporal information regarding the dynamics or – importantly – the direction of this process.

**Fig. 4:**
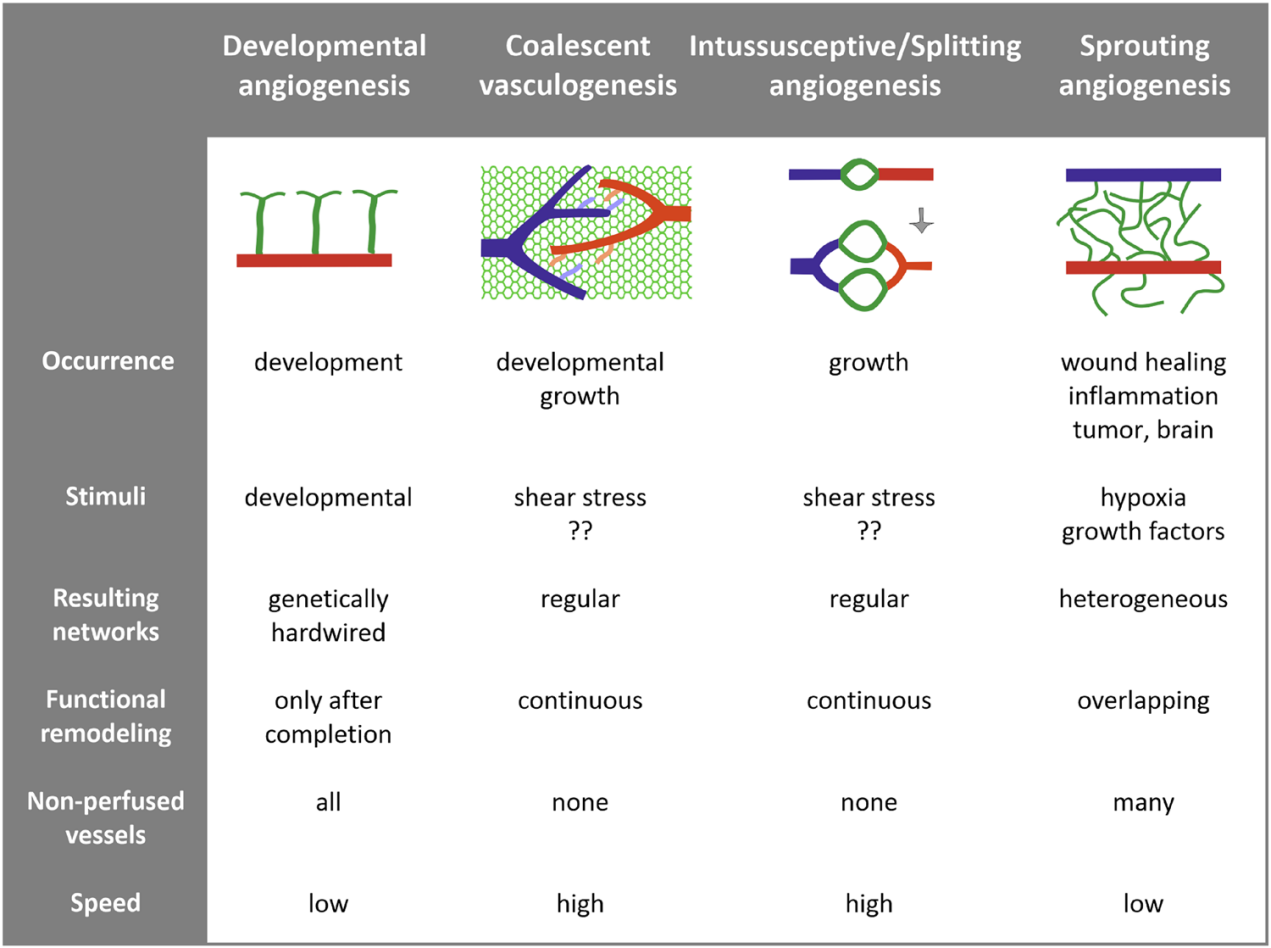
Characteristics of different patterns of microvascular growth. The newly described process of coalescent angiogenesis is compared to classic concepts of vascular sprouting and angiogenesis by vessel division (splitting or intussusceptive angiogenesis)

Continuous or repeated observation over periods relevant for angiogenetic processes can resolve this knowledge gap and as such, provide new insights into angiogenic modes and mechanisms. In the present study, the repeated observation of the microcirculation in the chick chorioallantoic membrane (CAM) yields evidence for a novel mode of angiogenesis which we termed *coalescent angiogenesis* (CA). In CA, blood vessel tree extension occurs via parallel processes of vessel maturation and regression of capillary mesh segments. As such, CA in the CAM of embryonic chicken eggs adds a novel mode of microvascular growth to previously described patterns of angiogenesis (Fig. 4).

These different modes are adapted for specific functional requirements and conditions. In *developmental sprouting angiogenesis*, vascular structures are formed de novo during embryonic development. The corresponding stimuli are generated by a highly conserved sequence of events, leading to a largely genetically determined vasculature. A typical example of developmental sprouting is the generation of the main vessels in the embryonic zebra fish [42]. The angiogenetic stimuli leading to *reactive sprouting angiogenesis* are generated in the postembryonic phase in response to pathophysiological and physiological events, including wound healing, inflammation, cancer, and muscle training [43, 44]. Due to the stochastic processes involved, resulting vascular structures are quite heterogeneous and require intensive vascular adaptation and pruning to obtain functionally adequate vascular networks [25].

Both forms of sprouting angiogenesis initially generate vessels with non-connected free ends which do not contribute to perfusion until they connect to a respective draining or feeding vessel. In contrast, intussusceptive (splitting) and coalescent angiogenesis allow the fast expansion of vascular beds while maintaining perfusion, tissue function, and permanent functional remodeling. Due to the continuous perfusion of the vascular bed, angiogenetic signals derived from local hemodynamics are likely to play a more central role in intussusceptive (splitting) and coalescent angiogenesis, as compared to hypoxia-derived signals characteristic for sprouting angiogenesis [39, 45, 46]. This specific mode of predominant regulation by hemodynamic rather than hypoxic cues may be particularly prominent in organs where lack of perfusion does not cause hypoxia such as the CAM and the lung.

*Intussusceptive angiogenesis* was first described by Burri et al*.* in the pulmonary vasculature and describes the division of existing vessels, leading to two parallel segments and thus to relatively uniform vessel networks [8, 9]. The central element in intussusceptive (splitting) angiogenesis is the intravascular formation of tissue pillars which fuse to generate a permanent wall between the new parallel vessels. Referred to as an intussusceptive pillar, this endothelial-lined intravascular structure is commonly not visible by light microscopy but has been studied largely by corrosion casting and scanning electron microscopy [16, 41, 47].

*Coalescent angiogenesis* shares many properties with splitting angiogenesis, albeit with a very different morphological process for vessel generation. CA starts from an isotropic capillary network which is transformed to give rise to hierarchical and functionally adequate arterial and venous vessel trees. This process not only remodels existing vessels with respect to their diameter and wall composition but generates novel vascular structures required for effective perfusion. In this process, capillaries of the isotropic mesh fuse to establish feeding or draining vessels via the elimination of tissue islands separating them. In this process, the tissue island attains a morphological appearance that resembles the tissue pillars seen in splitting angiogenesis (Fig. 3B and Supplemental Material 2). However, in coalescent angiogenesis, these structures are progressively eliminated over time to give rise to definitive vessels, while in intussusceptive (splitting) angiogenesis, they grow and fuse to split an existing vessel. Accordingly, coalescent angiogenesis may be seen as the inverse process to splitting angiogenesis.

In the past, analysis of non-sprouting angiogenesis has mainly focused on splitting or intussusception. However, it is important to note that the respective studies are almost exclusively based on static images which cannot discriminate vessel division from vessel fusion. This dilemma was recently highlighted in a review by Gifre-Renom and Jones who proposed vessel fusion as a mode of non-sprouting vascular adaptation [48]. In this context, they point out the principal difficulty to distinguish fusion processes (combination of two or more small vessels to a larger one) from intussusceptive angiogenesis (splitting of a large vessel into two or more smaller ones). Individual micrographs representing a single point in time show anatomical features which could fit to both processes: The small roundish or ellipsoidal structures seen in microvessels, e.g., close to branching points (see Figures 1E and 3A), may represent tissue islands in the process of elimination during ‘coalescent angiogenesis’ or tissue pillars which grow to separate an existing vessel in ‘splitting angiogenesis’.

Only by following the anatomical development at a given location over time, as done in the present study, coalescent angiogenesis by vascular fusion can be clearly separated from splitting angiogenesis (intussusception). For the vascular bed studied, this approach provides direct evidence for coalescent angiogenesis of established vessels as the mechanism for vessel enlargement. Future time series studies are needed to (re-) define the respective roles of coalescent angiogenesis and splitting angiogenesis in other tissues and conditions.

Coalescent angiogenesis seems to have partial similarities with intussusceptive arborization. Intussusceptive arborization segregates the various vessel generations of capillary mesh networks by formation of vertical pillars in rows and narrow tissue septa formed by pillar reshaping and pillar fusions segregate the new vascular entities. Also the formation of horizontal pillars and folds which separate the new vessels from the capillary plexus is described as intussusceptive arborization to form hierarchical microvascular segments restructuring the primitive capillary meshwork to a vascular tree [47]. In Phase II of coalescent angiogenesis, a rearrangement of tissue islands along the preferred pathway is observed along with the enlargement of tissue islands in adjacent regions in phase IV. However, in intussusceptive arborization, pillars and similar structures are generated de novo, whereas in coalescent angiogenesis they represent remnants of tissue islands. Further studies are needed to clarify these aspects.

The CAM is functionally a two-dimensional analog to the lung, with both structures exchanging oxygen and carbon dioxide with the exterior via a dense mesh of capillaries that are fed and drained by dichotomous hierarchical arterial and venous vessel trees, respectively. Thus, it is tempting to speculate that the mode of vascular growth observed here—i.e., coalescent angiogenesis—may also be relevant for the development and/or adaptation of the pulmonary circulation. Yet although lung intravital microscopy has been successfully established and advanced in several labs including ours, such studies pose a considerable technical challenge due to the complex three-dimensional structure of the pulmonary vasculature, the inherent problems associated with prolonged imaging of the lung, and motion artifacts over the respiratory and cardiac cycle [49, 50]. Even for the CAM model, the concept of coalescent angiogenesis requires further studies and in-depth analysis, e.g., with respect to the relevance of hemodynamic and metabolic stimuli, to the cellular and molecular mechanisms involved in the reorganization of vascular structures, as well as to the generation of the initial capillary mesh. The present results (Supplementry Figures S1 and S2) for regions remote from the CAM’s growing border zone indicate that capillary meshes are stable with respect to vessel area and length. However, in the late stages of coalescent angiogenesis (see Supplementary Material 2), the capillary mesh is reconstituted on top of the main vessels which slightly sink into the yolk sac.

There is evidence that angiogenesis in adult lungs is generally limited, except in pathological conditions such as tumor growth, wound healing, or fibrosis. With respect to tumor growth, there is a non-angiogenic process described as vessel co-option, which is an alternative mechanism of generating the blood supply that differs fundamentally from the well-known sprouting angiogenesis and occurs in a significant fraction in highly vascularized organs like lung, brain, and colon [51–55]. Here tumor cells grow into pre-existing mature blood vessels independently of stimulating new blood vessel formation. Vessel co-option, as well as vascular mimicry presents non-angiogenic processes that have been directly visualized in glioblastomas by intravital imaging [56]. Coalescent angiogenesis and vessel co-option are processes of neo-angiogenesis which leads to the fast generation of new blood vessels to increase the transport of oxygen and nutrients independently of angiogenic stimuli. In contrast to the pathological process of vessel co-option, coalescent angiogenesis is a physiological process which does not need invasive tumor cells. Several previous studies have highlighted the usefulness of the CAM model for the study of tumor invasion and metastasis [57, 58].

In summary, our study provides evidence for a novel mode of vascular network development, termed *coalescent angiogenesis*, that develops functional vascular trees from the initial homogeneous capillary mesh of the chick chorioallantoic membrane. Vessel generation by this mode may play an important role whenever organs with a pre-existing capillary mesh exhibit fast growth (e.g., liver, lung, pancreas). This hypothesis for a new mode of vascular maturation should be further investigated in future studies, including continuous observation as well as mechanistic and molecular analyses.

## Supplementary Information

Below is the link to the electronic supplementary material.

Supplementary file1 (DOCX 2974 kb)

Supplementary file2 (AVI 29127 kb)

Supplementary file3 (AVI 30196 kb)

Supplementary file4 (AVI 29736 kb)

## Data Availability

Data and image processing algorithms or code will be made available on request.

## Abbreviations

CAM: Chorioallantoic membrane
CA: Coalescent angiogenesis
ED: Embryonic development
FOV: Field of view
HH: Hamburger-Hamilton
IA: Intussusceptive angiogenesis
IAR: Intussusceptive arborization
IBR: Intussusceptive branching remodeling
IMG: Intussusceptive microvascular growth
ROI: Regions of interests
SA: Sprouting angiogenesis
SD: Standard deviation
VEGF: Vascular endothelial growth factor
