# Supplementary material for: Coalescent angiogenesis—evidence for a novel concept of vascular network maturation
Source: Angiogenesis. 2021 Dec 14;25(1):35–45. doi: 10.1007/s10456-021-09824-3 (PMC8669669; doi:10.1007/s10456-021-09824-3)
Supplement: Supplementary file 1 — Supplementary file1 (DOCX 2974 kb) [file 10456_2021_9824_MOESM1_ESM.docx]

**Supplementary Material 1: Video sequences**

Links:

**Movie 1:**

[**https://www.dropbox.com/s/ugqm51lov69h8jz/Nitzsche%20et%20al_%20Supp_Movie%201.avi?dl=0**](https://www.dropbox.com/s/ugqm51lov69h8jz/Nitzsche%20et%20al_%20Supp_Movie%201.avi?dl=0)

**Movie 2:**

[**https://www.dropbox.com/s/g8ebd6fwyli9r4u/Nitzsche%20et%20al_%20Supp_Movie%202.avi?dl=0**](https://www.dropbox.com/s/g8ebd6fwyli9r4u/Nitzsche%20et%20al_%20Supp_Movie%202.avi?dl=0)

**Movie 3:**

[**https://www.dropbox.com/s/auqdvwzxg65kgys/Nitzsche%20et%20al_%20Supp_Movie%203.avi?dl=0**](https://www.dropbox.com/s/auqdvwzxg65kgys/Nitzsche%20et%20al_%20Supp_Movie%203.avi?dl=0)

Representative movies of CAM vasculature. **Movie 1** shows CAM vascular network recorded by intravital microscopy. Red blood cell movement is seen in larger vessels of the arterial and venous side of the vascular bed. In **Movie 2,** the vessel tree, consisting of an arteriole (~30 µM in diameter) and venules with diameters in a range from 20, 40 and 80 µM, is connected to the capillary bed. Venous side branches are formed and perfused immediately. Capillary bed is only visible due to the movement of the red blood cells within the capillaries. Cells of the vessel walls are hidden within the opaque tissue. **Movie 3** shows the flow of erythrocytes through an arteriole and the capillary bed. Differences in red blood cell velocity are obvious and show preferred pathways of the blood cells. All videos have a resolution of 640 x 480 pixels and a video length of 10 s with a framerate of 50 fps.

**Supplementary Material 2: Networks**

SD images from different CAMs were selected showing one large venular branch which undergoes branching remodeling with tissue islands disappearing over time. The images are a supplement to Figure 3B.


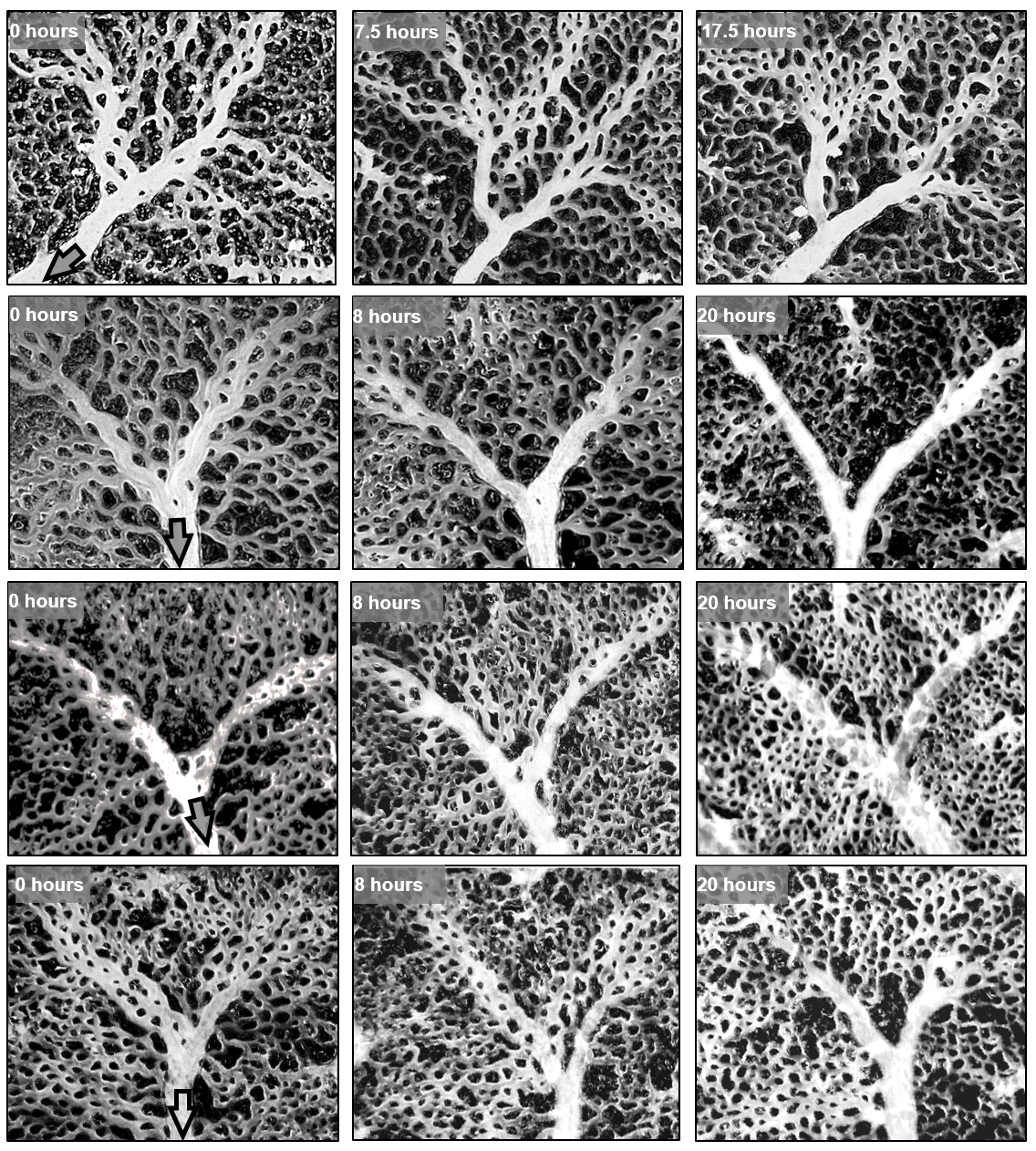


**Supplementary Material 3: S-Figures**


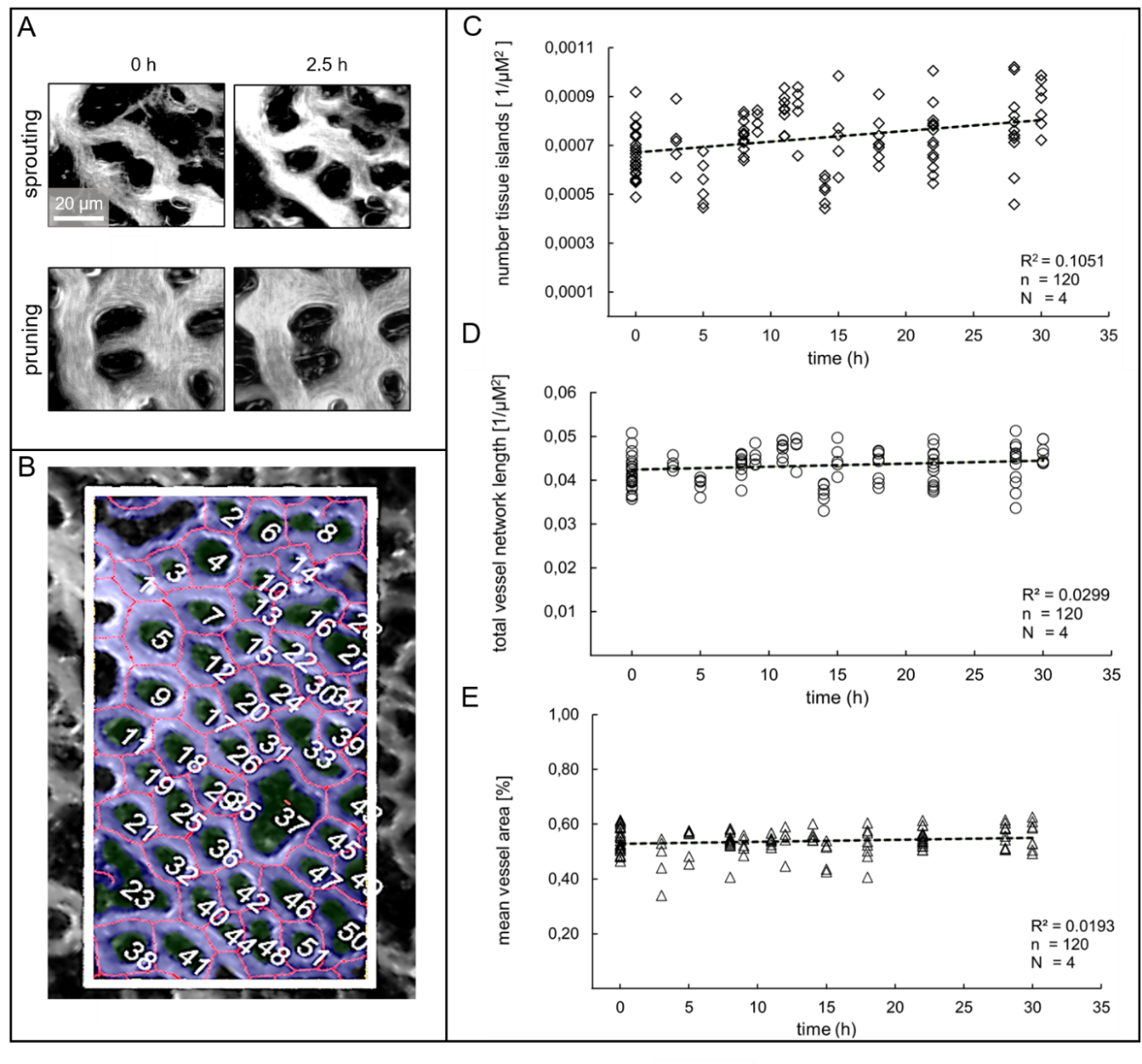


**S-Figure 1. A.** Dynamics of individual tissue islands. Upper panel: a tissue island is divided by sprout formation. Lower panel: fusion of two tissue islands by vessel pruning. Both mechanisms occur in parallel within the capillary bed of the CAM. **B-E.** Dynamics of mean morphological parameters. **B.** Region of interest (ROI) is demarcated by white frame. Vessel centerlines (red) are determined by automatic segmentation (as described in the method section of the manuscript). Tissue islands are determined and numbered consecutively. **C.** Number of tissue islands per area over 30 h shows a slight increase that does not reach the level of significance. **D, E.** Total vessel network length and mean relative vessel area are constant over time. The vessel network length is the sum of the vessel centerlines in the skeletonized images. Correlation coefficients R^2^ were calculated for 120 ROIs (n) from 4 CAMs (N).


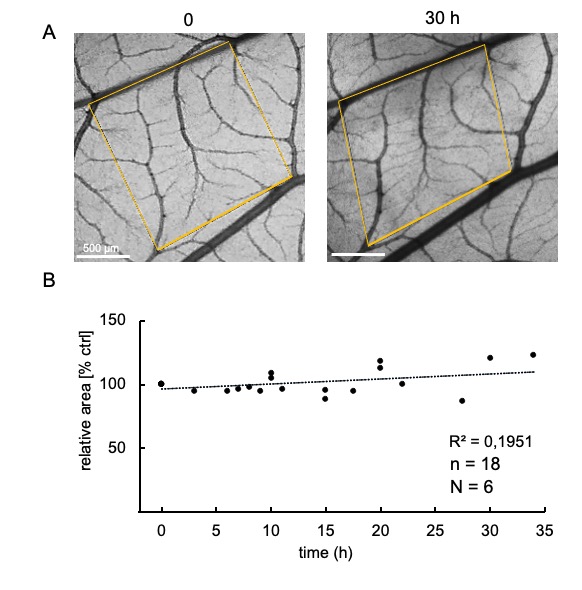


**S-Figure 2**. Dynamics of the CAM area. **A**. Region of interest (yellow quadrilateral) was traced over 33 h. The corners of the ROI were defined by topographical landmarks. **B.** Change in area over time recorded for 6 CAMs with three ROIs each, relative to the initial area.
